# Association of Inflammatory Profile During *Ex Vivo* Lung Perfusion With High-Grade Primary Graft Dysfunction: A Systematic Review and Meta-Analysis

**DOI:** 10.3389/ti.2025.13794

**Published:** 2025-01-29

**Authors:** Andrea Costamagna, Eleonora Balzani, Matteo Marro, Erika Simonato, Alessandro Burello, Mauro Rinaldi, Luca Brazzi, Massimo Boffini, Vito Fanelli

**Affiliations:** ^1^ Department of Surgical Sciences, University of Turin, Turin, Italy; ^2^ Cardiovascular and Thoracic Department, Azienda Ospedaliero Universitaria Città della Salute e della Scienza di Torino, Turin, Italy

**Keywords:** *ex vivo* lung perfusion, ischemia-reperfusion injury, primary graft dysfunction, inflammation, biomarkers

## Abstract

PGD3 is the manifestation of ischemia-reperfusion injury which results from inflammation and cell death and is associated with poor outcome. This systematic-review and meta-analysis of non-randomized controlled trials on patients undergoing Ltx with reconditioned lungs via EVLP, aims to assess the association between the levels of proinflammatory biomarkers during EVLP and PGD3 development within the firsts 72 h post-Ltx. Biomarkers were categorized by timing (1-hour, T0 and 4-hours, Tend from EVLPstart) and by their biological function (adhesion molecules, chemokines, cytokines, damage-associated-molecular-patterns, growth-factors, metabolites). We employed a four-level mixed-effects model with categorical predictors for biomarker groups to identify differences between patients with PGD3 and others. The single study and individual measurements were considered random intercepts. We included 8 studies (610 measurements at T0 and 884 at Tend). The pooled effect was 0.74 (*p* = 0.021) at T0, and 0.90 (*p* = 0.0015) at Tend. The four-level model indicated a large pooled correlation between developing PGD3 at 72 h post-Ltx and inflammatory biomarkers values, *r* = 0.62 (*p* = 0.009). Chemokine group showed the strongest association with the outcome (z-value = 1.26, *p* = 0.042). Pooled panels of inflammation markers, particularly chemokines, measured at T0 or at Tend, are associated with the development of PGD3 within the first 72 h after Ltx.

**Systematic Review Registration:**

https://osf.io/gkxzh/.

## Graphical Abstract

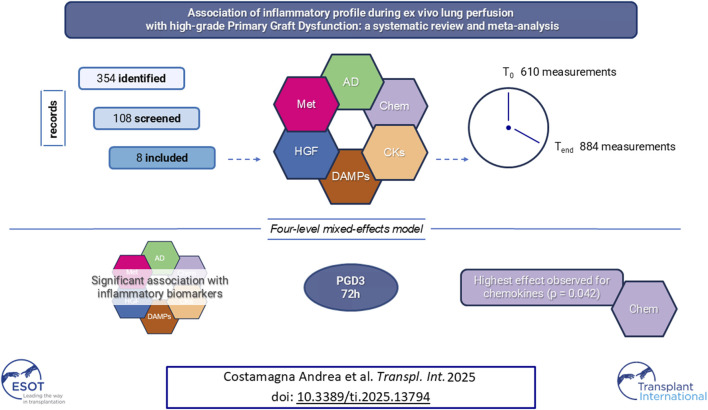

## Introduction

*Ex vivo* lung perfusion (EVLP) is a well-established platform to assess and potentially treat suboptimal donor lungs to mitigate organ shortage expanding the pool of lungs suitable for transplantation [1]. During 6 h of ventilation and perfusion, conventional clinical parameters such as respiratory mechanics, gas exchange, hemodynamics and radiologic appearance are monitored to assess organ suitability for transplantation. Additional measures and techniques have been proposed to enhance the accuracy of lung evaluation during EVLP, such as the application of machine learning models to radiographic findings [2], direct lung ultrasound assessment [3, 4] and tissue microdialysis for assessing lung tissue metabolism [5].

However, EVLP lungs may develop primary graft dysfunction (PGD), which is an early form of acute lung injury that occurs during the firsts 72 postoperative hours [6]. Severe form of PGD (namely, grade 3 PGD), characterized by PaO_2_/FiO_2_ < 200 mmHg, represents an established risk factor for poor recipients survival [7–9] and bronchiolitis obliterans development [6, 8].

PGD is the clinical manifestation of ischemia-reperfusion induced lung injury (IRI), which results from the complex interplay between inflammation, cell death and leukocyte activation in the donor lung [6, 10]. After lung procurement, damage-associated molecular patterns (DAMPs), such as HMGB1 and nuclear and mitochondrial DNA, are released as a consequence of cell death occurring during cold ischemic time and subsequent rewarming and reperfusion [11–15]. These endogenous molecules stimulate in turn a variety of immune (e.g., macrophages and lymphocytes) and non-immune cells (e.g., lung epithelial and endothelial cells and fibroblasts) to release pro-inflammatory mediators, such as chemokines and cytokines [10, 16]. Both pro-inflammatory mediators and DAMPS promote the expression of adhesion molecules, associated with endothelial activation and consequent leucocyte recruitment, leading to endothelial permeability increase with oedema formation [11, 17–19]. In addition, recent studies show that point of care protein assay based on IL6 and 8 cytokine levels in lung perfusate has a good accuracy in predicting recipients outcome [20]. To identify lung recipients who at higher risk of PGD 3 development after EVLP and to optimize their treatment, an appreciation for the effects of different classes of biomarkers measured in lung perfusate is relevant. The aim of this systematic review and meta-analysis is to evaluate the association between the levels of pro-inflammatory biomarkers collected at the start or at the end of the EVLP procedure and the development of PGD 3 in the Ltx recipient within the firsts 72 post-operative hours. We hypothesized that different classes of soluble mediators in lung perfusate would exert different effects on PGD3 development in lung transplant recipients.

## Materials and Methods

This systematic review and meta-analysis of randomized controlled trials (RCTs) and non-randomized controlled trials (NRCTs) was prepared in accordance with the Preferred Reporting Items for Systematic Reviews and Meta-Analyses (PRISMA) statement [21]. The Cochrane Handbook for Systematic Reviews of Intervention was chosen as the methodological guidance [22]. The protocol was registered in PROSPERO (PROSPERO #CRD42022296486).

The following databases were used: PubMed, Embase, and Scopus. Searches were conducted for studies published up to 03 August 2023. RCTs and NRCTs published in English, and Italian were considered eligible for inclusion. Other potentially relevant studies were searched in study registers (i.e., PROSPERO, ClinicalTrials.gov), and in gray literature sources. Specific search strategies were created for each database (Supplementary Figure S1).

Each step outlined by the PRISMA flow diagram, along with corresponding lists of included and excluded articles together with their respective justifications, can be found online.^1^

### Eligibility Criteria

The search was restricted exclusively to RCTs and NRCTs, encompassing participants who satisfied the subsequent inclusion criteria: adult human patients of all genders undergoing Ltx employing reconditioned lungs via EVLP.

The exclusion criteria were: investigations encompassing organ system care (OCS), and studies that reported inflammatory biomarkers at a timepoint preceding EVLP.

Patients who developed PGD with a P/F ratio < 200 mmHg along with radiographic lung infiltrates or requiring extracorporeal life support, are classified as having PGD 3 [23]. Considering the different impact on survival outcomes, we categorized our study population into those with severe PGD (PGD 3) and those with grade 1 or 2 PGD, or no PGD. The primary outcomes were biomarker levels measured in the perfusate at 1 h (T_0_) and 4 h (T_end_) from the start of EVLP. We selected all studies in which specific mediators of inflammation -e.g., Interleukin-8, Interleukin-6, etc.- were quantified in their genotypic or phenotypic expression related to the development of PGD 3 at 72 h.

### Selection Process

Search results were collated and exported to EndNote V.X9 (Clarivate Analytics, PA, United States). Duplicates were automatically removed. The review process consisted of two screening levels using Rayyan QCRI online software [24]:

(1) a title and abstract review
(2) full-text review

For both levels, 2 authors (AC and EB) independently screened the articles, with conflicts resolved by a third author (VF).

Data were extracted in a planned standardized Excel spreadsheet (*study characteristics, year of the study, type of population, biomarkers detected, timing of measures, and main results*). When the data were not directly available, we used WebPlotDigitizer or directly contacted the authors.

To prevent biased inclusion of data based on the results, the authors decided that 1) where trialists reported both final values and changes from baseline values for the same outcome, final values were recorded; 2) where trialists reported both unadjusted and adjusted values for the same outcome, unadjusted values were extracted; 3) where trialists reported data analyzed based on the intention-to-treat sample and another sample (e.g., per-protocol, as-treated), data from the former were extracted.

### Subgroup Analysis

Biomarkers were divided into subgroups basing on their biological function and mechanism of action in specific inflammatory pathway. Six categories were identified: adhesion molecules (sE-selectin, sICAM, vCAM, ET-1, Big ET-1), chemokines (IL-8, MCP, GROα, MIP-1a, MIP-1b), cytokines (IL1β, IL6, TNFα), damage-associated molecular patterns (M30, HMGB, nuDNA, mtDNA), growth factors (M-CSF, G-CSF) endogenous metabolites produced during inflammatory phenomena (CO and NOx).

### Study Risk of Bias Assessment

Two authors (AC and EB) independently assessed the risk of bias through the Risk Of Bias In NRCTs – of Interventions (ROBINS-i) tool [25]. RoB graph was created through RobVis visualization tool [26]. For NRCTs, an initial assessment considered potential confounders and co-interventions. Subsequently, the risk of bias was evaluated as either “low,” “uncertain,” or “high” in various domains, including confounders, participant selection, intervention classification, deviation from intended intervention, missing data, outcome measurement, and reported results. Any disagreements were resolved by a third reviewer (VF).

### Statistical Analysis

Since we assessed a continuous outcome (pro-inflammatory status) measured through different variables, we computed the standardized mean difference (SMD) along with its associated 95% confidence interval (95% CI). In the process of pooling the data, we employed a random-effects model and inverse variance method. To ensure robust performance in this analysis, we opted for the restricted maximum likelihood estimator (REML) for tau^2^. We used Q-Profile method for CI of tau^2^ and tau. We used the Hartung-Knapp adjustment for random effects model. The prediction interval was based on t-distribution. The calculation of SMD was carried out utilizing Hedges’ g method. The outcomes were represented using either forest plots or drapery plots, as suggested in the literature [27]. Prediction intervals were calculated and represented in the respective forest plots [28]. Statistical significance was set at *p* < 0.05. To evaluate the size of the effect of the SMD, we considered levels of 0.2, 0.5, 0.8 as small, medium, and large effects. Statistical heterogeneity was determined with the Q statistic and I^2^, with values of 25%, 50%, and 75% taken to indicate low, moderate, and high levels of heterogeneity, respectively [22]. To identify potential publication bias, the Egger’s regression test was performed.

Since study participants are nested within studies, we set a four-level meta-analysis to assess potential moderators of the overall effect using a four-level mixed-effects model. We included random effects for specific factors that could influence model reliability, such as the research group (level 4), individual authors (level 3 – nested within level 4), and the marker of interest (level 2 – nested within level 3). Level 1 represented individual measurements. As fixed effects, we incorporated the specific group of molecules (e.g., chemokines, cytokines, etc.), categorized based on their potential molecular mechanisms contributing to PGD, and Timing, which referred to the timing of individual measurements. Timing was defined as either immediately after the lung transplant subjected to EVLP or days later, categorized as T_0_ vs. T_end_. We used the REML method to estimate model parameters. To evaluate the model’s fit, we reported the Akaike Information Criterion (AIC). After establishing the suitability of the four-level model, we proceeded to assess potential moderators of the overall effect. The effect of individual biomarker categories was determined by adding the intercept value to their estimate [27].

We performed a sensitivity analysis using the same four-level meta-analysis, but restricted it to specific timepoints, namely, T_0_ and T_end_.

All the analysis were performed with R studio using the packages dmetar, meta, metafor version 2023.06.0+421 (2023.06.0+421) [29].

## Results

The search strategy retrieved 350 articles from databases and 4 from registers. After removing the duplicates, the remaining 108 articles were independently screened for titles and abstracts by 2 authors (AC and EB), and 96 records were excluded. A detailed list for each exclusion reason is available at link.^1^ Full texts of the remaining 12 records were screened, and 5 records were excluded. One last paper was added after a careful revision of the bibliography [30].

A total of eight NRCTs were included [12, 19, 20, 30–34] in the systematic review and meta-analysis. A detailed selection process is shown in the PRISMA flowchart (Supplementary Figure S1) and in a repository online.^1^ Then two authors (AC and EB) independently extracted the data following study protocol.

### Risk of Bias in Studies

**FIGURE 1:**
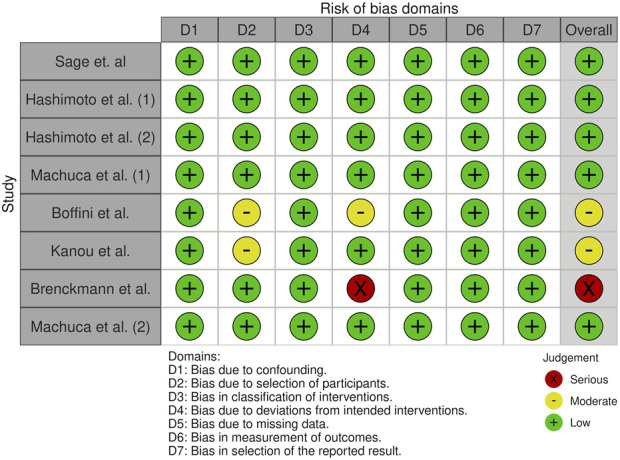
Risk of bias assessment using the Risk Of Bias In Non-randomised Studies - of Interventions (ROBINS-I) tool for the selected studies included in the meta-analysis.

All the studies included were judged of low RoB arising from the randomization process with ROBINS-I tool for NRCTs (Figure 1). The overall risk of bias was moderate-low due to the well-selected population, uniform protocols for detecting inflammatory mediators, and a low number of missing data. Potential confounders, such as the cutoff values for the measured variables and the kits used to detect the biomarkers, were identified.

### Description of Included Studies

A detailed description of the included studies is reported in Table 1.

**TABLE 1:** Summary of findings of the included studies.

Author	Year	Enrollment period	Country	Population characteristics	Biomarker evaluated	Exclusion criteria of the study	PGD 3 overall incidence
Sage et al.	2021	From 2009 to 2019	Canada	Not stated	• IL6 • IL8	Bilateral transplants that were not in the ICU or on ECMO support before transplant were excluded.	• PGD 3 at 72 h was 16%
Kanou et al.	2021	December 2018 to June 2019	Canada	39 % of the control group and 57 % of PGD 3 were DCD donors	• nuDNA • mtDNA	Lungs with hemorrhages.	• PGD 3 at 72 h was 52.6%.
Brenckmann et al.	2020	May 2010 and December 2015	France	45 % of the control group and 30 % of PGD 3 were DCD donors	• CO • IL6 • IL8 • IL1β • TNFα	Excluded 12 cases due to sample unavailability or brige to lung transplant with ECMO.	• PGD 3 at 72 h was 15.1%
Hashimoto et al.	2017	September 2008 to August 2013	Canada	62 % of the control group and 83 % of PGD 3 were DCD donors	• sICAM • sVCAM • sE-selectin	ECMO recipients before transplantation or who received single or lobar lung transplantation were excluded.	• PGD 3 at 72 h was 24%
Hashimoto et al.	2018	September 2008 to August 2013	Canada	38 % of the control group and 46 % of PGD 3 were DCD donors	• M30 • HMGB	ECMO recipients before transplantation or who received single or lobar lung transplantation were excluded.	• PGD 3 at 72 h was 24%
Machuca et al.	2015	September 2009 to November 2012	Canada	44 % of the control group and 43 % of PGD 3 were DCD donors	• M-CSF • G-CSF • MIP-1a • MIP-1b • IL8 • GROα	Single-lung transplants, lobar transplants, and recipients bridged to transplant with extracorporeal life support were not included in this study	• PGD 3 at 72 h was 15%
Machuca et al	2015	February 2009 to January 2010	Canada	48 % of the control group and 43 % of PGD 3 were DCD donors	• ET-1 • Big ET-1 • NOx	Bridge with ECMO and unilateral or bilobar transplants	• PGD 3 at 72 h was 22%
Boffini et al.	2023	July 2011 to March 2020	Italy	adsorption vs no adsorption population	• GCS-F • IL6 • MCP-1	Sample unavailability	• PGD 3 at 72 h was 23%

Abbreviations: Big ET-1: Big Endothelin-1; CO: Carbon Monoxide; ECMO: Extracorporeal Membrane Oxygenation; ET-1: Endothelin-1; GCS-F: Granulocyte Colony-Stimulating Factor; G-CSF: Granulocyte Colony-Stimulating Factor; GROα: Growth-Regulated Oncogene alpha Protein; HMGB: High Mobility Group B Protein; IL1β: Interleukin-1 beta; IL6: Interleukin-6; IL8: Interleukin-8; M30: Apoptosis Marker MCP-1: Monocyte Chemoattractant Protein-1; M-CSF: Macrophage Colony-Stimulating Factor; MIP-1a: Macrophage Inflammatory Protein-1 alpha; MIP-1b: Macrophage Inflammatory Protein-1 beta; mtDNA: Mitochondrial DNA; NOx: Nitrogen Oxides; nuDNA: Nuclear DNA; sE-selectin: Soluble E-selectin; sICAM: Soluble Intercellular Adhesion Molecule; sVCAM: Soluble Vascular Cell Adhesion Molecule; TNFα: Tumor Necrosis Factor alpha.

#### Biomarkers at Initial T_0_

**FIGURE 2:**
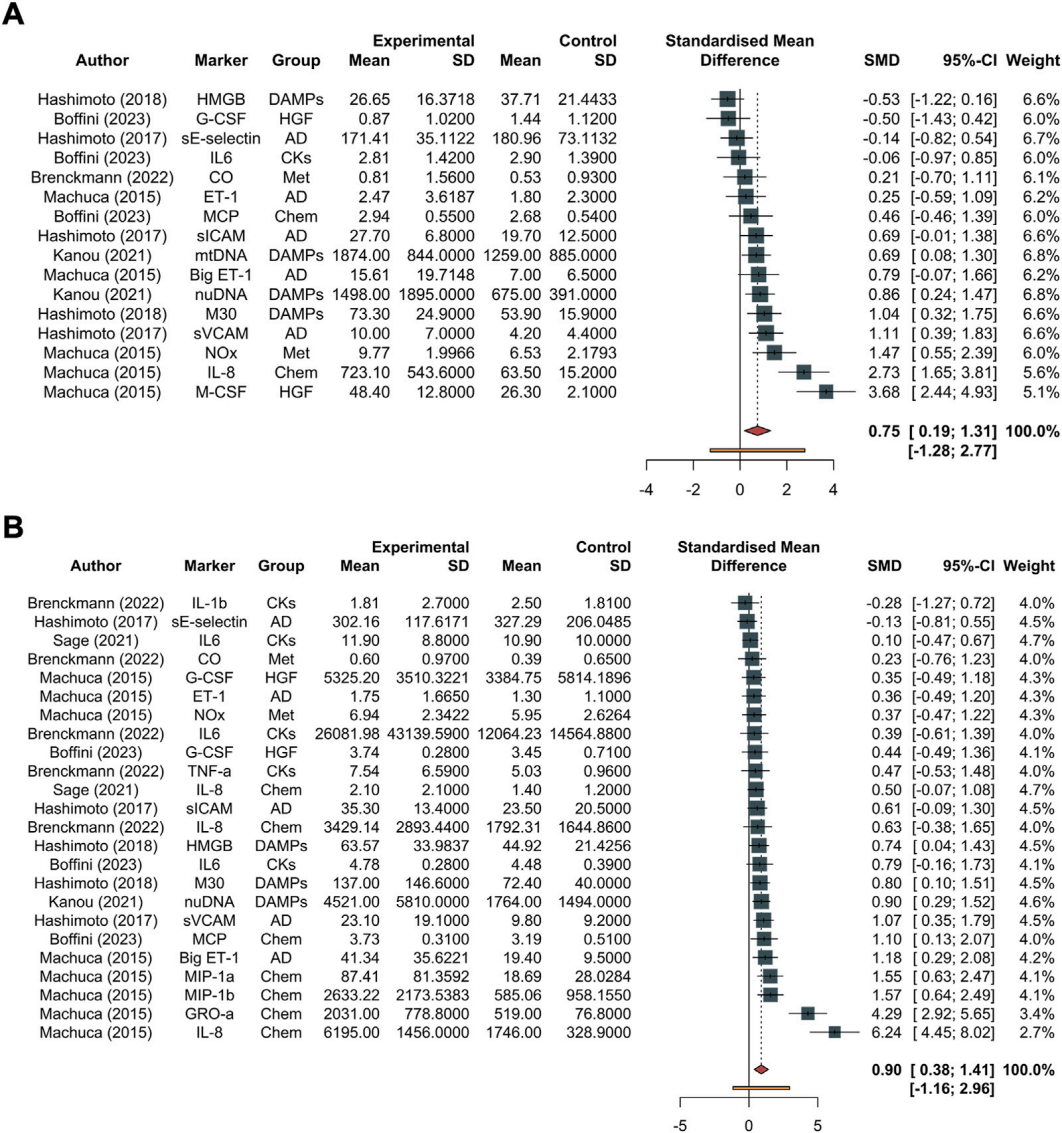
Forest plot of studies assessing inflammatory biomarkers. These are detailed in the column labeled “Marker,” accompanied by their corresponding groups denoted as “Group.” The experimental group corresponds to the PGD grade 3 at 72 h group, while the control group represents the non-PGD grade 3 group. The standardized mean difference (SMD), along with its respective 95% confidence interval (95% CI) and the individual weight for each study, is reported on the right. In the forest plot, squares placed to the right—considering 0 as the midpoint—indicate higher marker levels in the experimental group. **(A)** Forest plot for overall studies, excluding outliers, **(B)** all the studies. Abbreviations: AD, adhesion molecules; CKs, chemokines; DAMPs, damage-associated molecular patterns; Hematop GF, growth factors; sE-selectin, endothelial selectin; sICAM, intercellular adhesion molecule; vCAM, vascular cell adhesion molecule; ET-1, endothelin-1; Big ET-1, big endothelin-1; IL-8, interleukin-8; MCP, monocyte chemoattractant protein; GROα, growth-related oncogene alpha; MIP-1α, macrophage inflammatory protein-1 alpha; MIP-1β, macrophage inflammatory protein-1 beta; IL-1β, interleukin-1 beta; IL-6, interleukin-6; TNF-α, tumor necrosis factor alpha; M30, M30; HMGB, high mobility group box 1; nuDNA, nuclear DNA; mtDNA, mitochondrial DNA; M-CSF, macrophage colony-stimulating factor; G-CSF, granulocyte colony-stimulating factor.

In total, 610 measurements were conducted at the initial time. The inflammation mediators considered were sE-selectin, sICAM, vCAM, ET-1, Big ET-1, IL8, MCP, GROα, MIP1a, MIP1b, IL1β, IL6, TNFα, M30, HMGB, nuDNA, mtDNA, M-CSF, GCS-F, inhaled CO and NOx (Figure 2, and Supplementary Figure S2). The pooled effect according to the random-effects model was 0.75, with the 95% CI ranging from 0.19 to 1.31 (Figure 2 and Supplementary Figure S2). The effect size was 2.84 (*p* = 0.012), detecting a significantly higher probability to develop PGD grade 3 at 72 h post-transplant as the inflammatory biomarkers increase. The restricted maximum likelihood method estimated a between-study heterogeneity variance of *τ* ^2^, which was 0.82 with a 95% CI of (0.38–2.66) (Supplementary Table S1). The statistical heterogeneity (I^2^) was 78.6%, with a 95% CI of (66%–87%) and the significance test of Q (Q = 70, df = 15, *p* < 0.001) confirmed this heterogeneity (Figure 2). In the Eggers’ test, the intercept of our regression model was 4.66 (95% CI −0.67–9.98). This is non-significant (*t* = 1.714, p-value = 0.11), and indicates that the data in the funnel plot are quite symmetrical (Supplementary Figure S2).

#### Biomarkers at T_end_

In total, 884 distinct measurements were conducted at the final time. The inflammation mediators considered were sE-selectin, sICAM, vCAM, ET-1, Big ET-1, IL8, MCP, GROα, MIP1a, MIP1b, IL1β, IL6, TNFα, M30, HMGB, nuDNA, mtDNA, M-CSF, GCS-F, inhaled CO and NOx. The pooled effect according to the random-effects model was 0.90, with the 95% CI ranging from 0.38 to 1.41 (Figure 2, and Supplementary Figure S3). The effect size was 3.60 (*p* = 0.0015), detecting a significantly higher probability to develop PGD 3 at 72 h post-Ltx as the inflammatory biomarkers increase. The REML method estimated a between-study heterogeneity variance of *τ* ^2^, which was 0.93 with a 95% CI of (0.61–3.32) (Supplementary Table S1). The I^2^ was 74.9%, with a 95% CI of (62.7%–83.1%), and the significance test of Q (Q = 92, df = 23, *p* < 0.001) confirmed this heterogeneity.

**FIGURE 3:**
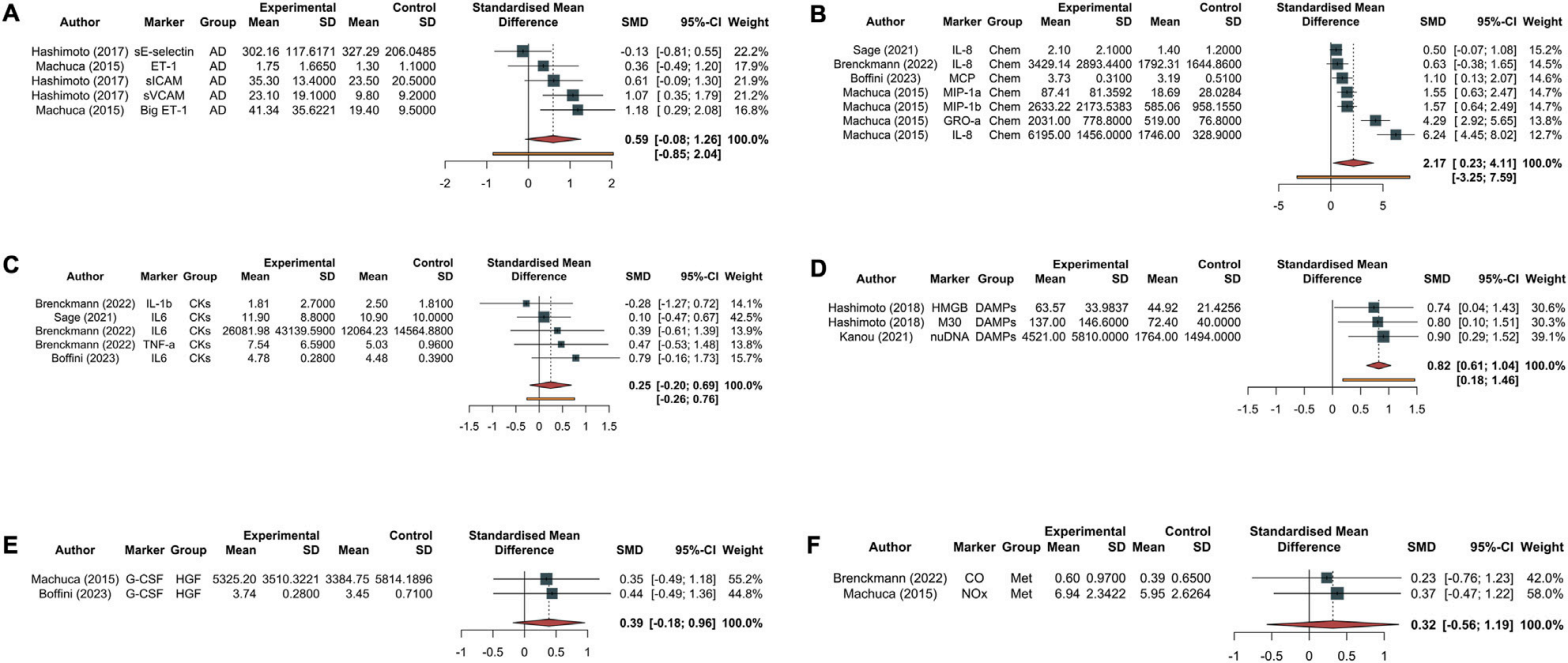
Forest plot of studies assessing inflammatory biomarkers at T_end_ corresponding to 4 h from EVLP start. Each plot represents a specific group of biomarkers: adhesion molecules [AD, **(A)]**, chemokines [Chem, **(B)**], cytokines [CKs, **(C)**], damage-associated molecular patterns [DAMPs, **(D)**], growth factors [HGF, **(E)**], and endogenous metabolites produced during inflammatory phenomena such as carbon monoxide and nitric oxide metabolite [Met, **(F)**]. The experimental group corresponds to the PGD grade 3 at 72 h, while the control group represents the non-PGD grade 3. The standardized mean difference (SMD), accompanied by its respective 95% confidence interval (95% CI) and the individual weight for each study, is reported on the right. In the forest plot, the placement of squares to the right of the plot—taking 0 as the midpoint—indicates higher marker levels in the experimental group. Abbreviations: sE-selectin, endothelial selectin; sICAM, intercellular adhesion molecule; vCAM, vascular cell adhesion molecule; ET-1, endothelin-1; Big ET-1, big endothelin-1; IL-8, interleukin-8; MCP, monocyte chemoattractant protein; GROα, growth-related oncogene alpha; MIP-1α, macrophage inflammatory protein-1 alpha; MIP-1β, macrophage inflammatory protein-1 beta; IL-1β, interleukin-1 beta; IL-6, interleukin-6; TNF-α, tumor necrosis factor alpha; M30, M30; HMGB, high mobility group box 1; nuDNA, nuclear DNA; mtDNA, mitochondrial DNA; M-CSF, macrophage colony-stimulating factor; G-CSF, granulocyte colony-stimulating factor; CO, carbon monoxide; NOx, nitric oxide metabolite.

In the Eggers’ test, the intercept of our regression model was 4.94 (95% CI 2.10–7.77). This is significant (*t* = 3.42, *p*-value = 0.025), and indicates that the data in the funnel plot are asymmetrical (Supplementary Figure S3). Single molecules are reported divided based on their category and the timing in Figure 3 (biomarkers at T_end_) and Supplementary Figure S4 (biomarkers at T_0_).

#### Four-Level Mixed Linear Analysis for Biomarkers Subgroups

We found a large pooled correlation based on the four-level meta-analytic model (*r* = 0.62–95% CI: 1.21–3.50; *p* = 0.009 - Table 2), meaning that there seems to be a substantial association between developing PGD 3 at 72 h post-Ltx and inflammatory biomarkers.

**TABLE 2:** Four-level multivariate meta-analytic model.

a. Random effect					
Factor	Variance	SD (sqrt)	Levels (nlvls)	Fixed	Variance (%)
Research group	0.0	0.0003	3	no	0.0
Research group + Author	0.304	0.5513	7	no	32.65
Research group + Author + Marker	0.6271	0.7919	26	no	67.35

b. Fixed effect		
Group	Odds ratio (OR with CI)	*p*-value
Chemochines	3.51 (1.05–11.79)	0.042
Cytokines	1.23 (0.30–5.05)	0.764
DAMPs	1.61 (0.34–7.69)	0.542
HGF	1.85 (0.43–8.04)	0.400
GroupMet	1.15 (0.23–5.71)	0.865
timing	1.21 (0.89–1.65)	0.213

Abbreviations: SD, standard deviation.

The estimated variance components were *τ* ^2^_Level2_ = 0.63, *τ* ^2^_Level3_ = 0.30 and *τ* ^2^_Level4_ = 0.00. This means that *I* ^2^_Level2_ = 67% of the total variation can be attributed to within-markers heterogeneity, and *I* ^2^_Level3_ = 33% to within-authors heterogeneity, while the within-research group heterogeneity was approximately *I* ^2^_Level4_ = 0%. Overall, this indicates that there is substantial between-study heterogeneity on the second level, or differences within studies. Yet, we also see that a large proportion of the total variance, more than one-fifth, can be explained by differences within markers (Level 3). In this model, the Akaike Information Criterion (AIC) of the four-level model was 109.7, indicating that the model fits the data well.

We checked if correlations differed depending on the biomarker group (AD, CKs, Chemokines, DAMPs, Hematop GF, Met) using a four-level moderator model (*p* = 0.235). In the model, the only group that showed a significant difference was the Chemokine group, with a z-value of 1.26 (*p* = 0.042). From our model, the timepoints were not associated with the outcome (OR 1.21 95% CI [0.89–1.65], *p*-value 0.213). We conducted a sensitivity analysis using data only at the timepoint T_0_ or at the timepoint T_end_, but the findings were consistent with the previous model (Supplementary Table S1).

## Discussion

In this systematic review and meta-analysis, soluble pro-inflammatory markers measured during EVLP were associated to PGD grade 3 at 72 h after transplantation. Chemokines levels exerted the highest effect on PGD development. Moreover, the timing at which these markers were collected did not affect their predictive value. In fact, patients with grade 3 PGD had higher biomarker levels measured at both early and late stages of EVLP.

The relationship between inflammatory molecules during IRI and the development of PGD is the consequence of the warm and cold ischemia following donor organ retrieval. Pro-inflammatory mediators can be activated during CIT as a result of oxidative stress, sodium Na^+^/K^+^ ATPase inactivation, calcium overload, and a variety of cell death mechanisms, including apoptosis, necrosis, autophagy, pyroptosis and ferroptosis [10, 35, 36].

Cellular stress triggers the release of DAMPs, mediating acute lung injury through TLR bonding. HMGB1 and nucleic acids were higher at T_end_ in the high-grade PGD patients [12, 33]; while the circulating amount of DNA was also higher at T_0_ in the PGD3 cohort, reflecting the amount of cell injury and death following CIT, HMGB1 levels were instead lower. In fact, HMGB1 can be actively released by alveolar macrophages, lymphocytes and epithelial cells too, after reperfusion, and maybe sustaining a pro-inflammatory effect in the damaged grafts [11–15]. High CKs levels (namely, IL1β, TNFα and IL6) at T_end_ might reflect lung injury, but in the examined studies the association between the total amount of these molecules and the development of PGD3 was not unanimous [20, 31, 32]. Hoffman et al. found no differences in terms of TNFα and IL1β in the plasma of LTx recipients with or without PGD3, and a significant increase in terms of IL-6 levels in patients with high grade PGD only 48–72 h after surgery [37]. This fact might corroborate the hypothesis that these CKs do not reflect the amount of tissue damage right after CIT. The included studies instead showed a strong association between chemokines levels in perfusate and PGD3, in particular when measured at the end of the procedure [20, 31, 32, 34]. Chemokines are involved in the recruitment of neutrophils (IL-8) and monocytes (MCP). IL-8 levels in particular are associated with worse graft function [10, 20, 37] and were also found to be higher at T_0_ in the recipients with worse outcome after transplantation [34]. Although G-CSF and M-CSF might theoretically play a role as the signature for a higher pro-inflammatory milieu, considered globally they were not strongly associated with PGD3 development, except for M-CSF levels in perfusate at T_0_ [31, 34] In fact, M-CSF is involved in monocytes and macrophages proliferation and differentiation [38], plays a role in the pathogenesis of pulmonary fibrosis [38] and has been associated with hyperinflammatory state in COVID-19 patients [39] Soluble adhesion molecules are released into circulation from the cell surface and their levels correlate with the degree of endothelial activation during inflammation [18, 19]. They are known to be upregulated in lung tissue samples from ARDS patients who died for Gram-negative bacteria induced septic shock [40] and in ARDS patients with worse outcome [17, 41, 42].Adhesion molecules were increased in every graft, except from sVCAM-1, which remained stable during the procedure only in those lungs which did not develop PGD3 [19]. Our metanalysis confirms that sVCAM-1 levels are constantly higher in the PGD3 recipients both at the beginning and at the end of EVLP. Moreover, the higher level of Big ET-1 in PGD3 grafts might rather reflect an alteration in capillary permeability and recruitment of inflammatory cells [30].

Compared to lungs that are transplanted without examination, EVLP provides a unique platform for dynamically assessing the inflammatory load. The molecules that can be retrieved in the perfusate might be the expression of a previous or ongoing biological damage or themselves sustain lung injury. A dynamic picture of the biological effects of the CIT and the degree of lung injury that occurred before organ retrieval may be obtained. Implementing the standard EVLP evaluation with a panel of biomarkers specific for the different stages of cell and tissue injury, might help in defining a peculiar biological signature for the single organ. This would be possible only with the availability of faster, standardised and reliable multi-parametric point of care tools. Lung assessment would then benefit from this upgrade under two perspectives: first, identifying - and discarding - organs with an unacceptable risk for early complications, such as high grade PGD and eventually death. Second, EVLP would become a platform for targeted treatments to a molecular level, to heal and recover lungs by acting on one or more mechanisms of the IRI injury cascade.

The Toronto Lung score is the only available and validated score that takes into consideration a point-of-care evaluation of pro-inflammatory CKs levels in EVLP perfusate; however, the analysis is restricted to IL6 and IL8 and does not account for the entire cascade that underlies lung injury [10, 17, 20]. This might not be enough. In fact, gene expression profiling on lung tissue has shown that whereas the inflammatory pathways are upregulated in DBD lungs, cell death, apoptosis, and necrosis predominate in the transcriptomic signature of DCD donors’ lungs [43].

This is the first study to summarise the current research about the predictive and prognostic role of IRI biomarkers measured during EVLP in a systematic and quantitative fashion. We employed a robust statistical method (namely - multilevel meta-analysis), which accounts for effect sizes. In addition, limiting the research to EVLP reduces the confounders that would have been generated including studies considering the donor lungs before the retrieval and/or in the recipient. In the first case the levels of the measured biomarkers are the result of a complex interaction between the multi-organ derangement following brain or cardiac death and of the interplay between lungs and the other organs. Additionally, because CIT has not yet occurred, its contribution to lung injury would not be measured. Conversely, the recipient faces the donor’s inflammatory load, together with the activation of the immune system and the effects of immune suppression, in a context of an end-stage pulmonary disease with potential multi-organic involvement. Another strength of this meta-analysis is that normothermic acellular EVLP is performed in few highly specialized centres in the world and even less ones collected and published their results. This guarantees that the procedures are homogenous, with similar learning curves and few deviations from the original protocols, making measurements highly comparable.

This study has some limitations. Firstly, the small number of studies limits the analysis to a pooled one and does not allow for molecule-by-molecule analysis. Therefore, the overall sample size is small and there is possible overlap of patients, because the same group might have measured different biomarkers on the same specimens. Secondly, it was not possible to compare directly across different studies due to their limited numbers. However, we mitigated this issue by using standardized mean differences to group molecules with similar biological significance, ensuring that all the biomarkers measure the same checkpoint in the inflammatory cascade but employing different scales [22]. Thirdly, the papers included comprise a long period of time and refer to specimen acquired in an even broader time span. In any case, normothermic acellular EVLP technology has not changed much over the past 15 years, and many studies refer to lung assessments performed from about the same period. Fourthly, we attempted to analyse the distribution of true effect sizes with the mean (*θ*_i,j_), considering all possible sources of heterogeneity. Despite conducting a meticulous analysis, we consistently observed high levels of heterogeneity, particularly between-studies. Considering the incorporation of prediction intervals in our results - providing a range to anticipate the effects of future studies based on current evidence - we posit that our findings might not attain statistical significance in the future, especially with the potential expansion of the sample or exploration of different biomarkers. This underscores the need to prioritize our focus on specific biomarkers. Fifth, most of the studies were of a retrospective nature, using prospectively collected materials, thus limiting the availability of data in terms of laboratory test results. Lastly, our prediction intervals, which can estimate between-study heterogeneity variance and the standard error of the pooled effect, suggest that our results may be subject to confirmation or revision by future studies, as the overall estimated effect is not statistically significant.

## Conclusion

Lung perfusate concentration of inflammatory biomarkers, in particular chemokines, are associated with Grade 3 PGD development at 72 h in recipients of EVLP lungs. Future multicentre, prospective observational studies are needed to confirm the results of this meta-analysis.

## Data Availability

The original contributions presented in the study are included in the article/Supplementary Material, further inquiries can be directed to the corresponding author.

## Footnotes

1
https://osf.io/gkxzh/

## Glossary

PGD: primary graft dysfunction
ICU: intensive care unit
Ltx: lung transplantation
**PaO**_**2**_: partial pressure of arterial oxygen
**FiO**_**2**_: fraction of inspired oxygen
IRI: ischemia reperfusion injury
CIT: cold ischemic time
DAMPs: damage-associated molecular patterns
EVLP: *Ex vivo* lung perfusion
**TLS**_**2**_: Toronto Lung Score 2
RCTs: randomized controlled trials
NRCTs: non-randomized controlled trials
PRISMA: Preferred Reporting Items for Systematic Reviews and Meta-Analyses
ROBINS-i: Risk Of Bias In Non-randomized Studies–of Interventions
sE-selectin: serum E selectin
sICAM: soluble intercellular adhesion molecule-1
vCAM: soluble adhesion molecules vascular cell adhesion molecule-1
ET-1: endothelin-1
Big ET-1: big endothelin-1
IL8: Interleukin 8
MCP: monocyte chemotactic protein
GROα: growth-regulated oncogene α
MIP-1a: macrophage inflammatory protein 1a
MIP-1b: macrophage inflammatory protein 1b
IL1β: Interleukin 1β
IL6: Interleukin 6
TNFα: tumour necrosis factor α
HMGB: High mobility group box 1
nuDNA: nuclear DNA
mtDNA: mitochondrial DNA
M-CSF: macrophage colony-stimulating factor
G-CSF: Granulocyte colony-stimulating factor
CO: carbon monoxide
NOx: nitric oxide metabolite
TLR: Toll-like receptor
