# Supplementary figures and images for: Association of Inflammatory Profile During Ex Vivo Lung Perfusion With High-Grade Primary Graft Dysfunction: A Systematic Review and Meta-Analysis
Source: Transpl Int. 2025 Jan 29;38:13794. doi: 10.3389/ti.2025.13794 (PMC11815944; doi:10.3389/ti.2025.13794)

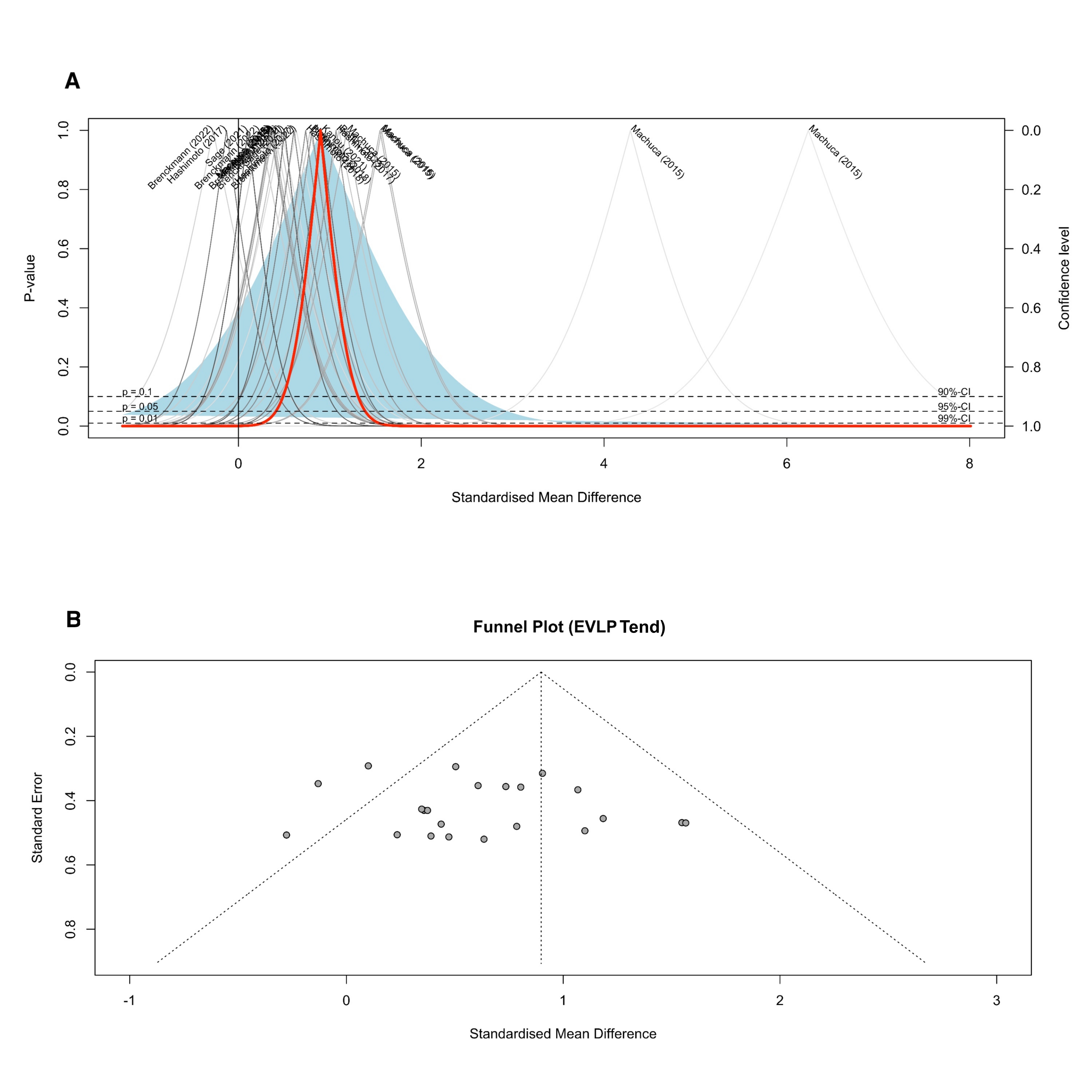

Supplement: Supplementary file 1 [file Image3.jpg]

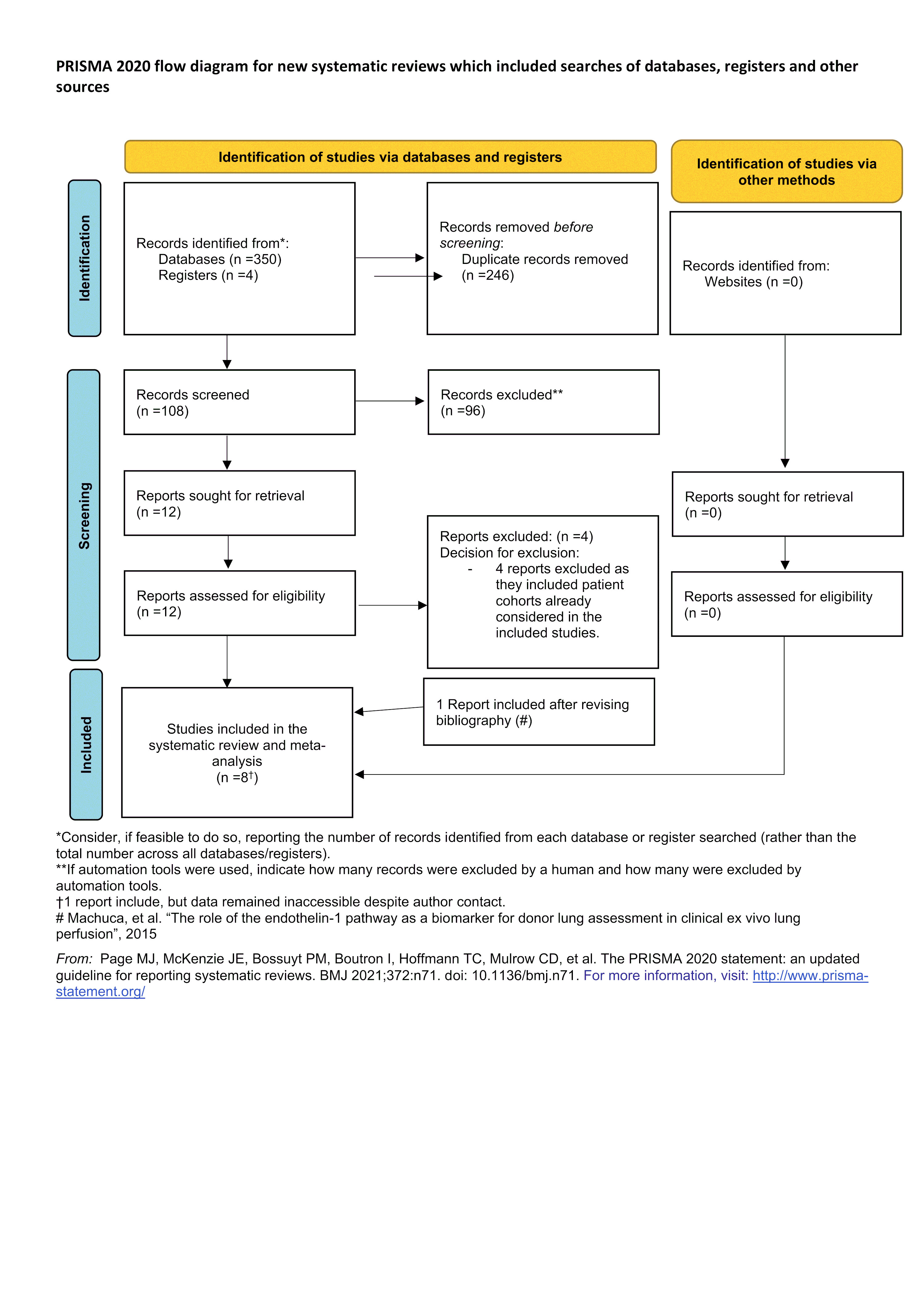

Supplement: Supplementary file 2 [file Image1.JPEG]

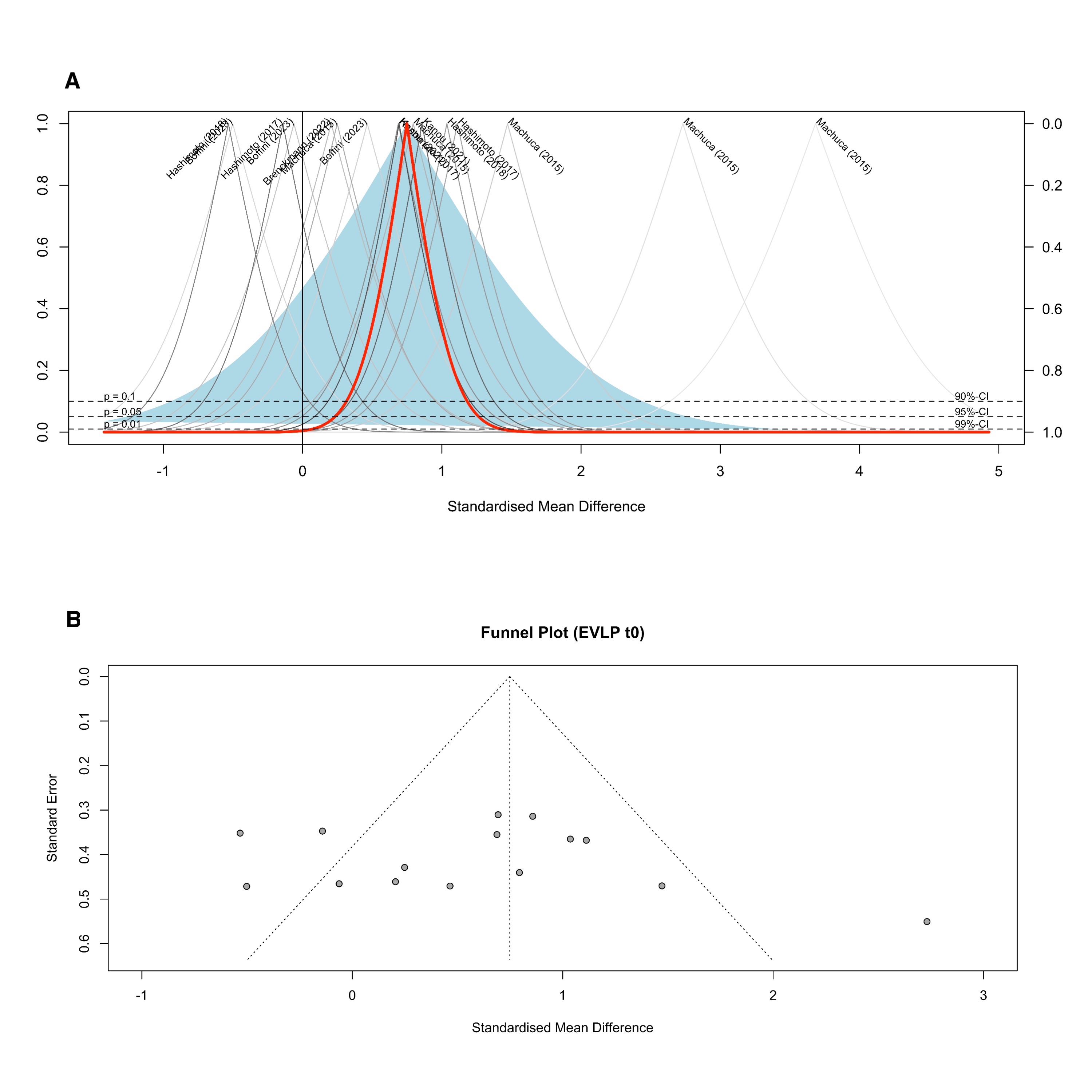

Supplement: Supplementary file 3 [file Image2.JPEG]

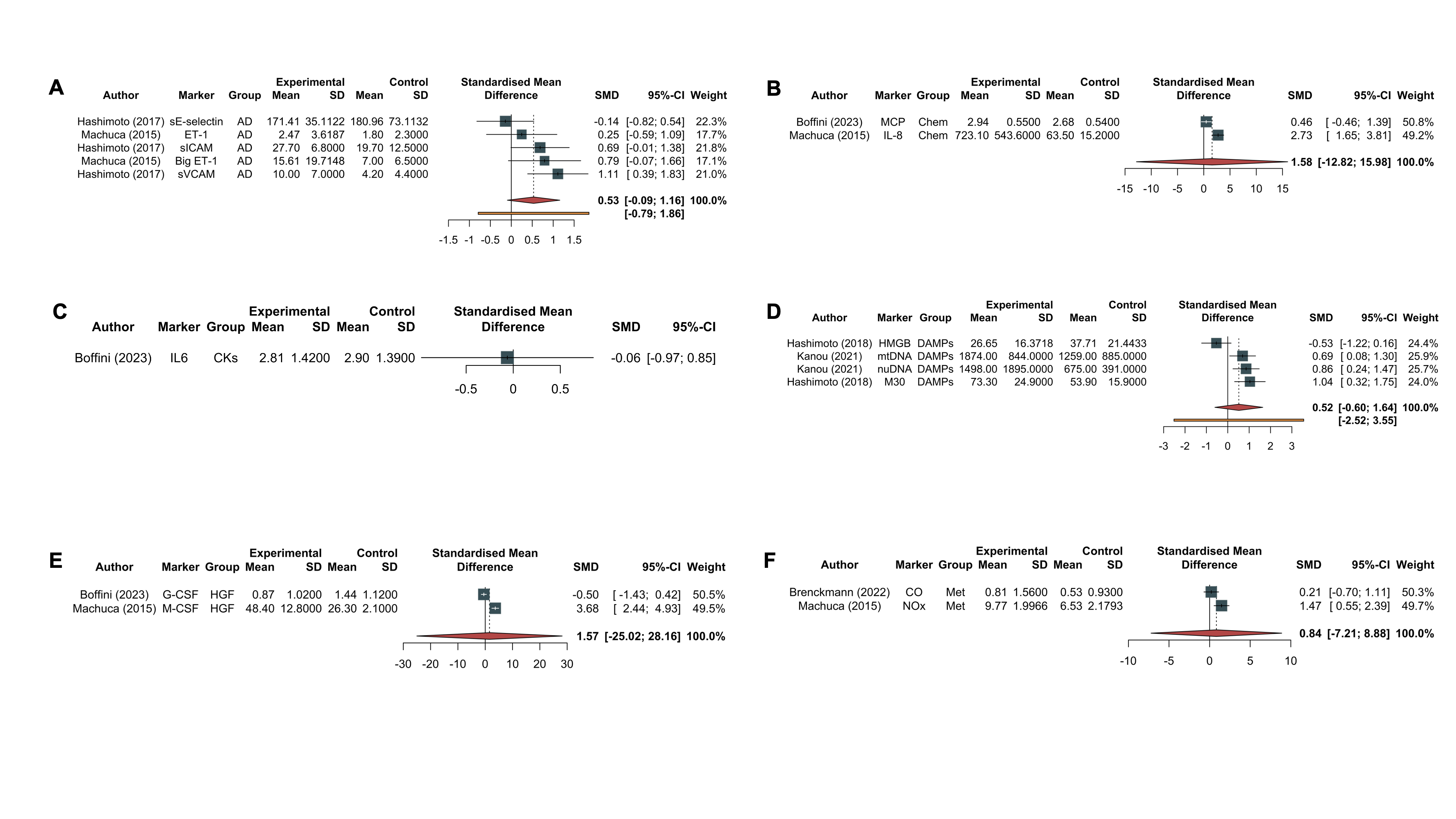

Supplement: Supplementary file 4 [file Image4.TIFF]
