# Supplementary material for: Association of Inflammatory Profile During Ex Vivo Lung Perfusion With High-Grade Primary Graft Dysfunction: A Systematic Review and Meta-Analysis
Source: Transpl Int. 2025 Jan 29;38:13794. doi: 10.3389/ti.2025.13794 (PMC11815944; doi:10.3389/ti.2025.13794)
Supplement: Supplementary file 5 [file DataSheet1.docx]

**Supplements**

**Figure S1.** Preferred Reporting Items for Systematic Reviews and Meta-Analyses (PRISMA) flow diagram, explaining the process at each stage of the systematic review.

**Figure S2.** Panel A: Drapery plot illustrating the standardized mean difference (SMD) at T0 (1 hour after EVLP start) between PGD grade 3 and No PGD grade 3 groups. Each study is represented by a confidence curve on the effect size (SMD) versus the assumed p-value. The 'peak' of the p-value functions precisely indicates the effect size in our meta-analysis. The x-axis denotes the effect size (SMD), and the y-axis represents the assumed p-value, with 90%, 95%, and 99% Confidence Intervals (CI) displayed. The thick line reaches zero on the x-axis when p is extremely small (<0.01), suggesting a high confidence in the pooled effect size being greater than zero.

Panel B: Funnel plot designed to visually assess publication bias in studies evaluating biomarker values at T0 (1 hour after EVLP start) between PGD grade 3 and No PGD grade 3 groups.

**Figure S3.** Panel A: Drapery plot illustrating the standardized mean difference (SMD) at T_end_ (4 hours after EVLP start) between PGD grade 3 and No PGD grade 3 groups. Each study is represented by a confidence curve on the effect size (SMD) versus the assumed p-value. The 'peak' of the p-value functions precisely indicates the effect size in our meta-analysis. The x-axis denotes the effect size (SMD), and the y-axis represents the assumed p-value, with 90%, 95%, and 99% Confidence Intervals (CI) displayed. The thick line reaches zero on the x-axis when p is extremely small (<0.01), suggesting a high confidence in the pooled effect size being greater than zero.

Panel B: Funnel plot designed to visually assess publication bias in studies evaluating biomarker values at \ (4 hours after EVLP start) between PGD grade 3 and No PGD grade 3 groups.

**Figure S4.** Forest plot of studies assessing inflammatory biomarkers at T_0_ corresponding to 1 hour from EVLP start. Each plot represents a specific group of biomarkers: adhesion molecules (AD, panel A), chemokines (Chem, B), cytokines (CKs, C), damage-associated molecular patterns (DAMPs, D), growth factors (HGF, E), and endogenous metabolites produced during inflammatory phenomena such as carbon monoxide and nitric oxide metabolite (Met, F).

The experimental group corresponds to the PGD grade 3 at 72 hours, while the control group represents the non-PGD grade 3. The standardized mean difference (SMD), accompanied by its respective 95% confidence interval (95% CI) and the individual weight for each study, is reported on the right. In the forest plot, the placement of squares to the right of the plot—taking 0 as the midpoint—indicates higher marker levels in the experimental group.

Abbreviations: sE-selectin, endothelial selectin; sICAM, intercellular adhesion molecule; vCAM, vascular cell adhesion molecule; ET-1, endothelin-1; Big ET-1, big endothelin-1; IL-8, interleukin-8; MCP, monocyte chemoattractant protein; GROα, growth-related oncogene alpha; MIP-1α, macrophage inflammatory protein-1 alpha; MIP-1β, macrophage inflammatory protein-1 beta; IL-1β, interleukin-1 beta; IL-6, interleukin-6; TNF-α, tumor necrosis factor alpha; M30, M30; HMGB, high mobility group box 1; nuDNA, nuclear DNA; mtDNA, mitochondrial DNA; M-CSF, macrophage colony-stimulating factor; G-CSF, granulocyte colony-stimulating factor; CO, carbon monoxide; NOx, nitric oxide metabolite.

**Table S1.** Multivariate meta-analytic model with restricted maximum likelihood estimation (REML) at both the initial and final time points (sensitivity analysis)

***1. Initial T_0_***

*a. Random effect*

| Factor | Variance | SD (sqrt) | Levels (nlvls) | Fixed | Variance (%) |
| --- | --- | --- | --- | --- | --- |
| Research group | 2.4486 | 1.5648 | 3 | no | 90.58 |
| Research group  + Author | 0.0 | 0.0 | 6 | no | 0.0 |
| Research group  + Author  +Marker | 0.2545 | 0.5045 | 16 | no | 9.42 |

Abbreviations: SD: standard deviation

*b. Fixed effect*

| Group | Odds Ratio (OR with CI) | p-value |
| --- | --- | --- |
| Chemochines | 12.36 (2.7 - 56.73) | 0.0043 |
| Cytokines | 9.65 (1.02 - 91.5) | 0.0485 |
| DAMPs | 0.98 (0.39 - 2.48) | 0.9705 |
| HGF | 10.96 (2.25 - 53.37) | 0.0071 |
| GroupMet | 2.59 (0.53 - 12.63) | 0.2096 |
| timing | 12.36 (2.7 - 56.73) | 0.0043 |

***2. Final T_end_***

*a. Random effect*

| Factor | Variance | SD (sqrt) | Levels (nlvls) | Fixed | Variance (%) |
| --- | --- | --- | --- | --- | --- |
| Research group | 0.0 | 0.0 | 3 | no | 0.0 |
| Research group  + Author | 0.173 | 0.4159 | 7 | no | 20.45 |
| Research group  + Author  +Marker | 0.673 | 0.8203 | 24 | no | 79.55 |

Abbreviations: SD: standard deviation

*b. Fixed effect*

| Group | Odds Ratio (OR with CI) | p-value |
| --- | --- | --- |
| Chemochines | 3.94 (1.1 - 14.07) | 0.0362 |
| Cytokines | 1.03 (0.24 - 4.31) | 0.9702 |
| DAMPs | 1.48 (0.29 - 7.55) | 0.6198 |
| HGF | 0.78 (0.14 - 4.45) | 0.7697 |
| GroupMet | 0.78 (0.14 - 4.46) | 0.7645 |
| timing | 3.94 (1.1 - 14.07) | 0.0362 |
